# Identification of associations between small molecule drugs and miRNAs based on functional similarity

**DOI:** 10.18632/oncotarget.9577

**Published:** 2016-05-24

**Authors:** Jing Wang, Fanlin Meng, EnYu Dai, Feng Yang, Shuyuan Wang, Xiaowen Chen, Lei Yang, Yuwen Wang, Wei Jiang

**Affiliations:** ^1^ College of Bioinformatics Science and Technology, Harbin Medical University, Harbin 150081, P. R. China; ^2^ The 2nd Affiliated Hospital, Harbin Medical University, Harbin 150081, P. R. China

**Keywords:** small molecule, miRNA, drug, functional similarity, microarray

## Abstract

MicroRNAs (miRNAs) are a class of small non-coding RNA molecules that regulate gene expression at post-transcriptional level. Increasing evidences show aberrant expression of miRNAs in varieties of diseases. Targeting the dysregulated miRNAs with small molecule drugs has become a novel therapy for many human diseases, especially cancer. Here, we proposed a novel computational approach to identify associations between small molecules and miRNAs based on functional similarity of differentially expressed genes. At the significance level of *p* < 0.01, we constructed the small molecule and miRNA functional similarity network involving 111 small molecules and 20 miRNAs. Moreover, we also predicted associations between drugs and diseases through integrating our identified small molecule-miRNA associations with experimentally validated disease related miRNAs. As a result, we identified 2265 associations between FDA approved drugs and diseases, in which ~35% associations have been validated by comprehensive literature reviews. For breast cancer, we identified 19 potential drugs, in which 12 drugs were supported by previous studies. In addition, we performed survival analysis for the patients from TCGA and GEO database, which indicated that the associated miRNAs of 4 drugs might be good prognosis markers in breast cancer. Collectively, this study proposed a novel approach to predict small molecule and miRNA associations based on functional similarity, which may pave a new way for miRNA-targeted therapy and drug repositioning.

## INTRODUCTION

MicroRNAs (miRNAs) are a class of small single-stranded non-coding RNA molecules which regulate gene expression by inducting cleavage or inhibiting translation of target mRNAs [1]. These small non-coding RNAs play crucial roles in many biological processes, such as proliferation, differentiation and apoptosis, and are related to varieties of disorders [1, 2]. Modulation of aberrantly expressed miRNAs has been demonstrated as a feasible strategy for many diseases [3]. At present, many kinds of miRNA modulators have been used in preclinical and clinical studies, such as antisense oligonucleotides (also known as anti-miRs), miRNA sponges, miRNA mimics [4–6]. Therefore, targeting dysregulated miRNA is a potential therapeutic regimen to develop miRNA specific drugs [7]. For example, SPC3649 (a kind of locked nucleic acid) is the first miRNA-targeted drug in clinical trials to inhibit miR-122 expression that is required by hepatitis C virus replication [8]. However, the major hurdles in oligonucleotides-based therapy are the properties of inefficient delivery and suboptimal pharmacodynamics or pharmacokinetics, which urgently need for small molecule-based intervention strategies [9]. Small molecules maybe suitable agents to regulate miRNA expression, because they are less expensive to produce, easily diffuse across cell membranes, easily delivered into cells as well as having good solubility, bioavailability, and metabolic stability [10]. For example, Bose *et al.* have identified the small molecule streptomycin, which is widely used for treatment of tuberculosis, as an inhibitor of miR-21 with a potential cancer therapeutic. Moreover, streptomycin could decrease miR-21 expression without affecting the expression levels of other related miRNAs [11].

Currently, a wide number of studies have devoted to develop high-throughput methods to screen small molecule modifiers of miRNAs, which may provide a new direction for miRNA-targeting therapies [4, 12–14]. Zhang *et al.* have presented the structure-based approaches, such as molecular docking, to screen compounds that targeting miRNAs [10]. In another structure-based method, Bose *et al.* reported a novel fluorescent molecular-beacon-based high-throughput method to screen small molecules which inhibited miRNA expression by blocking the Dicer processing [4]. Besides the structure based method, Jiang *et al.* proposed a novel method to construct small molecule-miRNA networks for 23 different cancers based on the similarity of transcriptional responses [15]. Similar work has also been done for Alzheimer's disease [16]. However, since lack of miRNA-transfected datasets, we simulated the transcriptional responses of miRNA perturbation through intersecting target genes and differential expressed genes of specific disease. Along with the growth of miRNA transfection experiments, it is possible to regard the effects of the miRNA on gene expression at whole genome level, which more directly reflects alteration of gene expression affected by the perturbed miRNA. What's more, miREnvironment and SM2miR database have been constructed to collect the experimentally supported associations between small molecules and miRNAs [17, 18], Currently, miREnvironment database have collected complex interactions (3857 entries) among 1242 miRNAs, 305 phenotypes and 394 environment factors (including a few small molecules). SM2miR database have collected the 5160 records between 255 small molecules and 1680 miRNAs, and provided the effects (up-regulated and down-regulated) of small molecules on miRNA expression, and integrated all associations between drugs and miRNAs in miREnvironment database.

**Figure 1:**
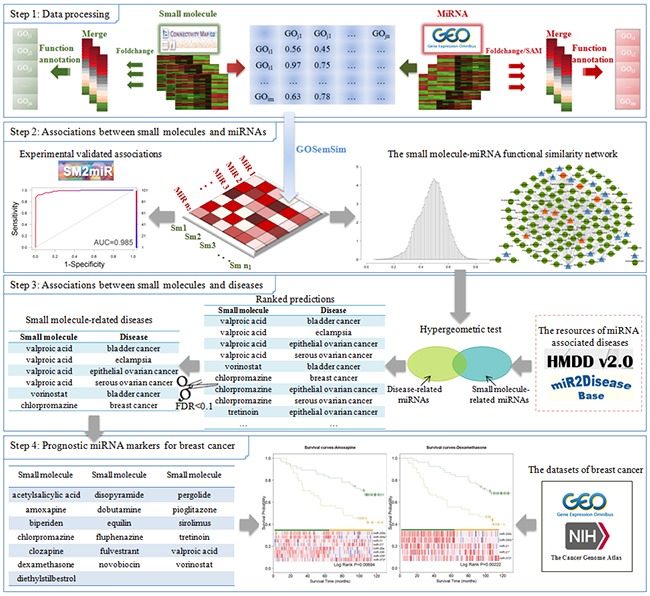
The workflow diagram of our approach

In this study, we proposed a novel approach to predict potential associations between small molecules and miRNAs based on functional similarity of differentially expressed genes of drug treatment and miRNA perturbation. In addition, through integrating the identified small molecule-miRNA associations with curated disease related miRNAs, we predicted drug-disease associations, which were used for drug repositioning. Finally, the miRNAs related to several predicted potential breast cancer drugs had the ability to distinguish patients with good or poor prognosis (the workflow diagram was shown in Figure 1). In a word, our method provides a novel prospect for developing miRNA-targeted drugs and predicting drug repositioning.

## RESULTS

### Small molecule-miRNA functional similarity network

**Figure 2:**
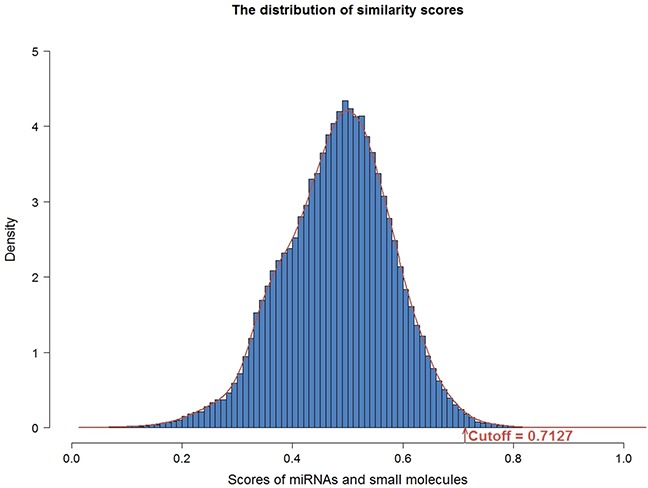
The distribution of functional similarity scores The similarity scores followed an approximate normal distribution with the mean and standard deviation equaling 0.4882 and 0.0965. The cutoff was determined by the value of the normal distribution at the significance level of 0.01, which was 0.7127.

**Figure 3:**
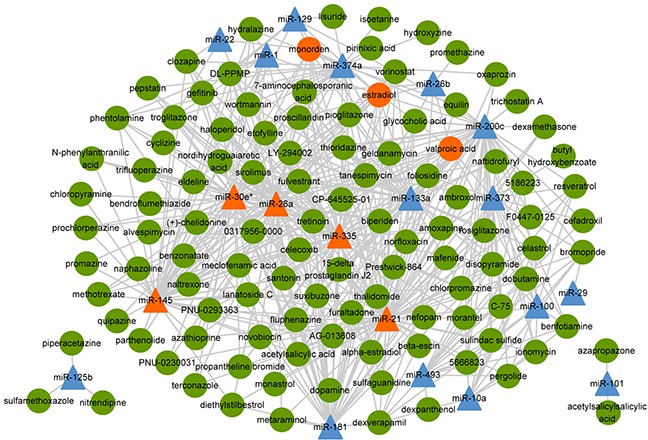
The small molecule-miRNA functional similarity network The triangles and circles represent miRNAs and small molecules, respectively. The orange triangles and circles are hub nodes of miRNAs (degree greater than 50) and small molecules (degree greater than 10), respectively.

In order to construct the functional similarity network between small molecules and miRNAs, we first collected gene expression profiles under perturbation of 88 miRNAs and treatment of 1309 bioactive small molecules. Second, we identified differentially expressed genes for small molecules and miRNAs. Then, we calculated functional similarity based on Gene Ontology (GO) enrichment analysis of the differentially expressed genes for each pair of small molecule and miRNA. As a result, we obtained the functional annotations of 1293 small molecules and 70 miRNAs (details in materials and methods). Here, similarity scores followed an approximate normal distribution with mean and standard deviation equaling to 0.4882 and 0.0965, respectively (Figure 2). The threshold of similarity score was determined by the value of the normal distribution at the significance level of 0.01, which was 0.7127. Finally, we constructed the small molecule-miRNA functional similarity network (538 associations) under the threshold of similarity score (0.7127), including 111 small molecules and 20 miRNAs (Figure 3, details in Supplementary Table S1). Especially, all 20 miRNAs in our predicted associations have been proved to play important roles in cancer, which were supported by previous studies. Taking miR-21 as an example, miR-21 is an oncogenic miRNA which is overexpressed in several tumors including breast cancer [19]. When suppressing the expression of miR-21 in breast cancer cells, cancer cells significantly reduced invasion and metastasis [20]. Its associated small molecule Tretinoin (the similarity score is 0.745) is also known as all-trans-retinoic acid, which is an important regulator of cell reproduction, proliferation and differentiation. In addition, Tretinoin is usually used to treat acute promyelocytic leukemia [21] and has been demonstrated to have potential therapeutic action in breast cancer [22]. Therefore, miR-21 may be a potential target for Tretinoin in the treatment of breast cancer.

Next, we explored the topological properties of this similarity network. As shown in Supplementary Figure S1a, most small molecules (~40%) connected with a small number of miRNAs (less than 20%). In Supplementary Figure S1b, most miRNAs (50%) connected with a small number of small molecules (less than 20%).

Furthermore, we determined the characteristics of small molecule pairs connecting with the same miRNA or miRNA pairs connecting with the same small molecule. First, we computed the Meet/Min score for each pair of miRNAs to evaluate the extent of shared target genes [15, 16]. The Meet/min score between two miRNAs was defined as the number of common target genes of two miRNAs divided by the small number of target genes of the two miRNAs. Our result showed that miRNA pairs connected with the same small molecule had moderate target similarity (*p* = 0.1892). Second, we employed the two-dimensional Tanimoto chemical similarity score, which was measured by Small Molecule Subgraph Detector (SMSD) software [23], to evaluate the structural similarity between small molecules. The findings indicated that the small molecule pairs connecting with the same miRNA had moderate structure similarity (*p* = 0.1223). All above results were consistent with the previous study [24].

**Figure 4:**
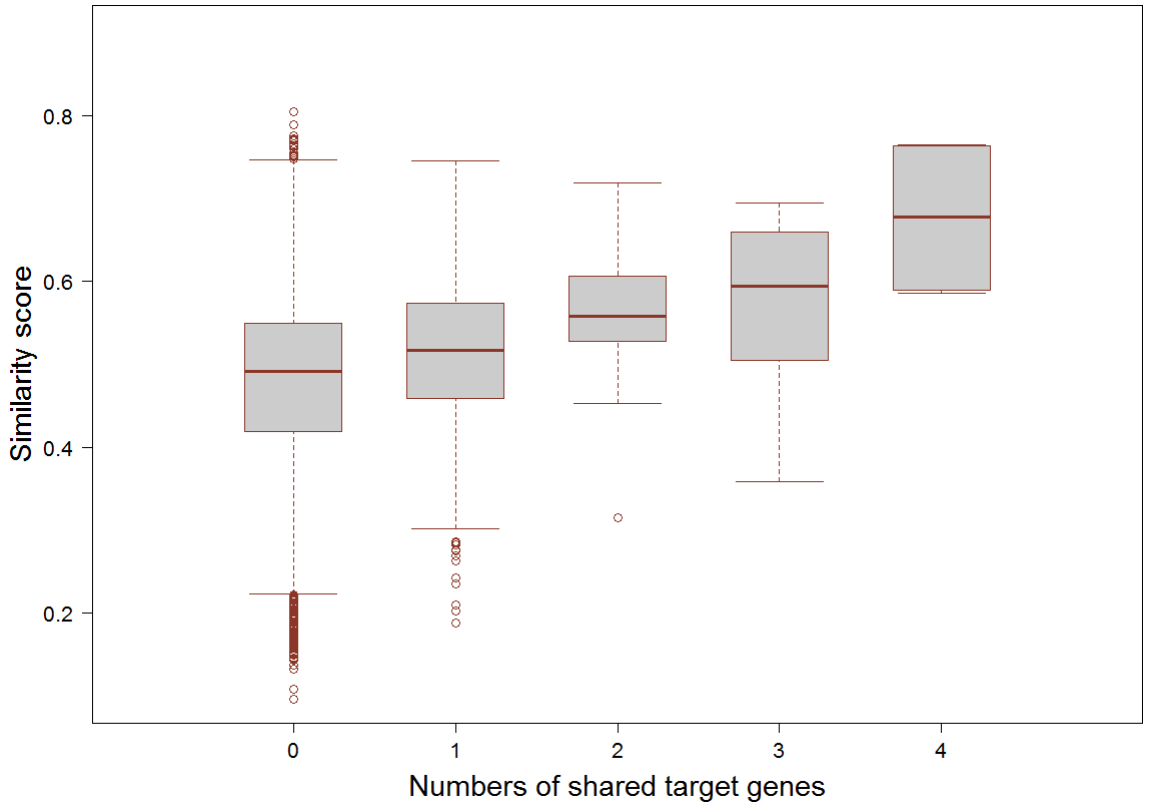
The relationship of the numbers of common target genes and the functional similarity scores The x-coordinate is the number of shared target genes between FDA-approved small molecule and miRNA, and the y-coordinate is the similarity score between the pair of small molecule and miRNA.

In addition, we further checked the potential relationship between functional similarity and the number of common targets of small molecule-miRNA pairs. We collected FDA approved drugs and their target information from the DrugBank database (http://www.drugbank.ca/), which included 624 small molecule drugs and 378 drug targets intersected with our dataset [21]. Meanwhile, we obtained the experimentally validated miRNA target genes from miRecords (Release 4.0) [25], miRTarBase (Release 4.5) [26], and TarBase (Release 6.0) [27]. Through integrating these regulations, we finally got 16510 miRNA-target gene pairs for 70 miRNAs in our study. For each association between FDA approved small molecule drug and miRNA, we counted the common target genes and compared with the similarity scores of these associations (Figure 4). The results demonstrated that the association with the higher similarity score tended to have the larger number of common target genes (Spearman's rank correlation *r* = 1, *p* = 0.017).

### Evaluation of the identified associations between small molecules and miRNAs

**Figure 5:**
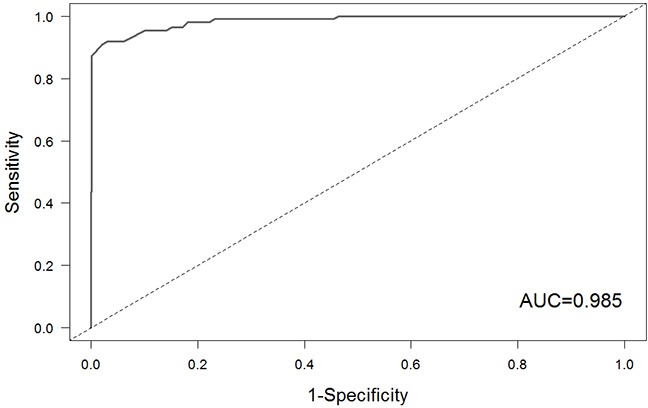
The ROC curve of our method based on the gold standard set

The receiver-operating characteristic (ROC) curve was employed to evaluate the performance of our approach base on the known small molecule-miRNA associations from SM2miR database (http://bioinfo.hrbmu.edu.cn/SM2miR/). First, we obtained 110 associations between small molecules and miRNAs in SM2miR database, which also appeared in this study. The 110 associations were considered as gold standard set. For each association in the gold standard set, we randomly selected the same number of GO terms of the miRNA and recalculated the similarity between the randomly GO terms and the GO terms of small molecule. This procedure was repeated 99 times and produced 99 fake functional similarity scores for each association. Considering the associations in the gold standard set as the positive instances and fake associations as negative instances, we calculated the sensitivity and the specificity of our approach at different cutoffs and plotted ROC curve (Figure 5). The area under roc curve (AUC) was 0.985, which suggested that the gold standard set indeed ranked on the higher position among 11000 pairs (110 true associations and 10890 fake associations). This result indicated that our approach achieved good performance in prediction of associations between small molecules and miRNAs.

### Prediction of drug indication based on small molecule-miRNA associations

The small molecule and miRNA functional similarity network could be used to predict drug repositioning. First, we collected experimentally validated disease related miRNAs from miR2disease [28]and HMDD (version 2.0) [29] databases. Then, we integrated these two databases and obtained 8134 associations between miRNAs and diseases in total. All redundant associations were removed via converting the similar disease names into MeSH unique IDs (http://www.ncbi.nlm.nih.gov/mesh). For the 20 miRNAs in the identified small molecule-miRNA functional similarity network, we obtained 506 miRNA-disease associations, involving 192 diseases. Combining the predicted 538 small molecule-miRNA associations in this study, we identified statistically significant small molecules-diseases pairs based on shared miRNAs by hypergeometric test. At the cutoff of adjusted *p*-value (FDR) < 0.1, 2265 pairs between 50 FDA-approved drugs and 155 diseases were identified. Among them, in 53 pairs, the drugs have already been used for treatment of the corresponding disorders, and ~35% (779/2265) pairs, involving 43 FDA-approved drugs and 128 diseases, have been supported by *in vitro* or *in vivo* experiments in previous studies. For example, the association of acetylsalicylic acid and colorectal cancer was significant in our prediction (*p*=0.006, FDR=0.061). Acetylsalicylic acid (also known as aspirin) is a common drug for treatment of mild to moderate pain and clinically indicated in the treatment of others disorder, such as arterial and venous thrombosis [21]. The effectiveness of Acetylsalicylic acid in regulating growth and differentiation of cancer, such as colorectal cancer, has also been reported *in vitro* and *in vivo* experiments [30–33]. Thus, the predicted potential drug indications based on the common miRNAs of small molecules and diseases might be used for drug repositioning.

### Identification of prognosis marker miRNAs for breast cancer

In this study, 19 small molecule drugs were predicted to associate with breast cancer. 12 out of 19 small molecule drugs have been supported in previous studies (Table 1). In order to investigate whether miRNAs that were associated with these small molecules could stratify patients into different prognosis groups, we collected datasets including miRNA expression and clinical information from The Cancer Genome Atlas (TCGA) and Gene Expression Omnibus (GEO) database. In TCGA database, we obtained 465 breast cancer samples with miRNA expression and clinical information. All miRNA expression profiles were processed and calculated for RPM (reads per million). In GEO database, we obtained one dataset with enough samples (GEO accession number: GSE19783, 99 patients). All miRNA expression was quantified using Agilent Human miRNA Microarray 2.0. For each small molecule drug, we firstly extracted expressions of miRNAs associated with this small molecule. And then, we classified all patients into two groups using K-means cluster method (K=2) based on these miRNAs expression.

**Table 1:** Predicted small molecule-disease associations in breast cancer

small molecule	*p*-value	FDR	PMID
Chlorpromazine	0.022865	0.081112	--
Dobutamine	0.00804	0.062879	--
Acetylsalicylic acid	0.022865	0.081112	24945997
Dexamethasone	0.013933	0.070355	25556455
Diethylstilbestrol	0.013933	0.070355	25278253
Equilin	0.013933	0.070355	Treatment
Fluphenazine	0.022865	0.081112	3695509
Fulvestrant	0.022865	0.081112	25876901
Novobiocin	0.00804	0.062879	20039369
Pioglitazone	0.022865	0.081112	23959881
Sirolimus	0.023168	0.081467	24099044
Tretinoin	0.022865	0.081112	23602051
Valproic acid	0.000677	0.036818	20159363
Vorinostat	0.023168	0.081467	24903226
Amoxapine	0.00804	0.062879	--
Biperiden	0.009832	0.062879	--
Clozapine	0.020443	0.077678	--
Disopyramide	0.003099	0.052926	--
Pergolide	0.001621	0.046908	--

Note: “Treatment” indicates that the small molecule is a FDA approved drug for treatment of breast cancer. “--” represents that the associations haven't verified by literatures. The red records represent four small molecule drugs that their associated miRNAs could stratify patients into good and poor prognostic groups both in TCGA and GEO database.

**Figure 6:**
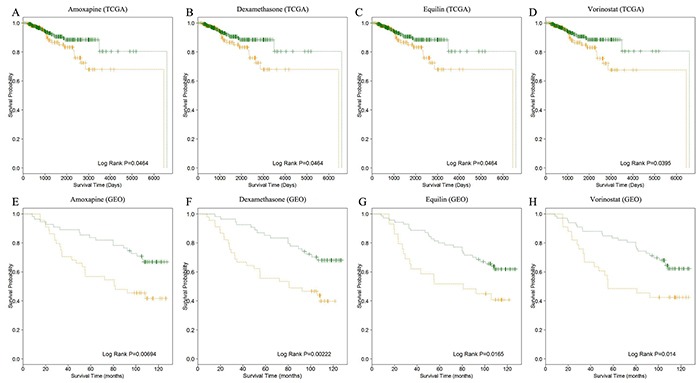
The survival analysis of breast cancer patients **A, B, C, D.** shows the Kaplan–Meier curves of good (in green) and poor (in orange) prognosis groups based on the TCGA datasets. Analogously, **E, F, G, H.** shows the Kaplan–Meier curves prognosis of good (in green) and poor (in orange) prognosis groups for the four small molecule drugs based on GEO datasets.

Finally, we discovered that 19 and 4 small molecule-related miRNAs could classify the breast cancer patients into high-risk and low-risk groups significantly (log-rank test p-value < 0.05) in TCGA and GEO datasets, respectively (Figure 6 and Supplementary Figure S2). The four small molecule drugs were marked red in Table 1. It was observed that miR-200c had associated with all of the four small molecules and tended to up-regulated in good prognosis groups. Analogously, we also found that miR-26a was related to two of the four small molecule drugs and tended to be up-regulated in good prognosis groups (Table 2). In breast cancer, miR-200c and miR-26a have been proved to be aberrantly expressed [34–37]. MiR-200c, the predominant member of the miR-200 family, could inhibit migration, invasion and cell polarization cancer-related processes [36]. MiR-26a has been reported as tumor suppressor miRNA and inhibited proliferation and migration through repression of MCL-1 (an anti-apoptotic member of the Bcl-2 family) in breast cancer [37]. These findings proved that the miRNAs related to small molecule drugs may be an efficient strategy for predicting the prognosis of diseases.

**Table 2:** The fold change of miR-200c and miR-26a expression between good and poor prognosis groups (TCGA and GEO)

Datasets	MiRNA	Amoxapine	Dexamethasone	Equilin	Vorinostat
TCGA	miR-200c-3p	1.270	1.270	1.270	1.274
	miR-200c-5p	1.062	1.062	1.062	1.060
	miR-26ac-3p	1.832	--	--	1.815
	miR-26ac-5p	1.113	--	--	1.116
GEO	miR-200c-3p	1.076	1.130	1.123	1.051
	miR-200c-5p	1.455	1.813	1.623	1.183
	miR-26a-5p	1.027	--	--	1.053

Note: “--” represented that the small molecule drug was not related to the miRNA in our methods.

## DISCUSSION

In this study, we successfully identified the associations between small molecules and miRNAs through calculating the functional similarities of their perturbed genes. We firstly collected the miRNA perturbed gene expression profiles from GEO database. In previous studies, because of lacking the miRNA transfection datasets, we simulated the dysregulated genes of miRNA perturbation through intersecting miRNA's target genes and differentially expressed genes of one disease. Along with the miRNA transfection datasets, the effects of the miRNA on gene expression at the whole genome level more directly reflect the alteration of gene expression affected by miRNA. Meanwhile, we obtained the small molecule perturbed gene expression profiles from the Connectivity Map (cMap, build 02) database [38]. These expression profiles reflect the transcription response to small molecules rather than the ‘putative target’ that the small molecule can be modulating. Based on the transcription responses perturbed by small molecule, some research focusing on broader themes of mechanism of action (MoA) elucidation [39, 40], drug repositioning [41, 42] and biological understanding [43, 44] have been performed. Moreover, we selected the transcription profiles of cell lines treated by small molecules and miRNAs to screen the perturbed genes. The functional similarity score for each pair of small molecule and miRNA was finally calculated based on the GO annotations of their perturbed genes.

Consequently, we identified 538 associations between 111 small molecules and 20 miRNAs. What's more, we obtained the curated small molecule-miRNA associations from SM2miR database as gold standard set to evaluate the performance of our approach. As a result, the AUC was 0.985. Furthermore, we identified small molecule-disease associations based on shared miRNAs, which provided a new way to predict the potential drug indications. Finally, survival analysis based on expression of miRNAs related to small molecule drugs for breast cancer revealed that these miRNAs might be good prognosis markers.

Some studies have devoted to predict the associations between small molecules and miRNAs. For example, *Chen et al.* predicted novel disease-related interactions between environmental factors (such as drugs) and miRNAs through a semi-supervised classifier based method [45]. Meng *et al.* proposed a novel method to predicted candidate small molecule-miRNA associations based on the similarity of transcription responses [46]. In this study, our approach tended to identify relationships between small molecules and miRNAs based on functional associations. Comparing with the transcription responses, functions were usually more reproducible.

In conclusion, we presented an approach to identify associations between small molecules and miRNAs based on functional similarities, which might provide a valuable perspective for repurposing drugs and predicting novel drug targets.

## MATERIALS AND METHODS

### Gene expression profiles under miRNA perturbation

We collected gene expression profiles from the GEO database by keywords “miRNA transfection” or “microRNA transfection”. We only reserved datasets with both miRNA transfected samples and control samples. We obtained 110 gene expression profiles of miRNA mimics (mimic endogenous miRNAs) or inhibitors (inhibit endogenous miRNAs) transfection in multiple species. We only chose 92 miRNA perturbed gene expression profiles (excluding gene expression profiles perturbed by multiple miRNAs or edited miRNAs) in human for further analysis, which involved 88 miRNAs (see Supplementary Table S2).

For single channel gene expression profiles with greater than or equal to two samples in each sample class (“transfection” or “control”), we applied significance analysis of microarray (SAM) [47] to identify significantly differentially expressed genes (FDR<=0.05). Otherwise, we identified the differentially expressed genes by 2-fold change method. For double channel gene expression profiles, 2-fold change was also used to measure the extent of differential expression of probes in each sample. Then, the conflict genes (gene appeared both in up-regulated and in down-regulated sets) were discarded. Only genes that were up-regulated or down-regulated in more than half samples were defined as differentially expressed genes.

Because some gene expression profiles measured under the same miRNA perturbation, we should combine the differentially expressed genes in these datasets. If the genes were up-regulated or down-regulated consistently in more than 2 datasets, the genes were considered differentially expressed under the miRNA perturbation.

### Gene expression profiles under small molecule treatment

We collected the small molecule perturbed gene expression profiles from the cMap. The cMap contains 6100 gene expression profiles of 1309 bioactive small molecules treatment and the corresponding controls. The differential expression was measured by amplitude (*A*), which was defined as:

$$A=\frac{t-c}{\frac{1}{2}\left(t+c\right)}$$

where *t* is the expression value of the probe in treatment group, *c* is the expression value in the control group. We defined that the probes with more than 2-foldchange between treatment and control group as the differentially expressed probes. Namely, the *A* value more than 2/3 means up-regulation, while the *A* value less than −2/3 means down-regulation.

Several gene expression profiles were measured under the treatment of the same small molecule, so we should combine the differential expressed genes of the same small molecule. Analogous to the differentially expressed genes of miRNA, only genes that were up-regulated or down-regulated in at least two expression profiles were considered as the differentially expressed genes.

### Functional enrichment analysis of small molecules and miRNAs

For the differentially expressed genes of miRNA (or small molecule), we implemented function enrichment analysis by the R package GOSim [48]. The version of GOSim package that we used in this study was 1.2.7.7. Biological Process (BP) was selected as the annotation category. Here, we chose the “elim” method, which improved the enrichment analysis via removing the genes mapped to significant GO terms from all their ancestors and calculates the significance of these GO terms using Fisher's exact test [49]. At the significance level of *p* < 0.01, we identified functions of 1293 small molecules and 70 miRNAs.

### Functional similarity between small molecule and miRNA

For each pair of small molecule and miRNA, we calculated the functional similarity score based on the annotated GO function. We implemented the computational procedure of similarity analysis with the R package GOSemSim [50]. The version of GOSemSim that we used was 1.20.0. The GOSemSim presented five methods, including Resnik [51], Jiang [52], Lin [53], Schlicker [54]and Wang [55], to calculate the semantic similarity of two given GO terms and four measures, including max, avg, rcmax and BMA, to combine semantic similarity scores of multiple GO terms. Here, we chose the widely used “Lin” and “BMA” methods. “Lin” method determined the semantic similarity of two given GO terms based on the annotation information content (*IC*). The *IC* of a GO term denoted that the negative logarithm of probability of the term appearing in the GO corpus.

The probability of a given GO term *t* is defined as:

$$p\left(t\right)=\frac{n_{t}}{N}$$

where *n_t_* is the number of its all children nodes plus itself, and *N* is the total number of terms in GO corpus. The *IC* is defined as:

IC(t)=−log(p(t))

The Lin method is defined as:

$$\text{sim}\left(t_{1},t_{2}\right)=\frac{2\text{IC}\left(\text{MICA}\right)}{\text{IC}\left(t_{1}\right)+\text{IC}\left(t_{2}\right)}$$

where *IC*(*MICA*) is the information content of their closest common ancestor (*t*_1_ and *t*_2_), the method normalized similarity between two given terms ranging from 0 to 1. The similarities among two sets of GO terms could organize as a matrix. The “BMA” measurement used the best-match average strategy to calculate the average of all maximum similarities on each row and column. Here, we selected the “BMA” method to compare the semantic similarity between two GO term sets.

### The targets of miRNAs

Previous studies indicated that a combination of multiple algorithms might increase the reliable of miRNA targets [56]. Thus, we obtained miRNA targets from seven prediction algorithms as previous study [57], including DIANA-microT [58], miRanda [59], RNA22 [60], RNAhybrid [61], TargetScan [62], miRBase Targets [63] and Pictar [64]. Only miRNA targets that were predicted in at least two algorithms were considered as reliable. In total, we obtained 289469 miRNA regulations for 776 miRNAs.

### Chemical structure similarity between two small molecules

In order to calculate structure similarity between two small molecules, we download the SDF file from Pubchem database (https://pubchem.ncbi.nlm.nih.gov/) [65]. The small molecules without structure information were filtered out. The two-dimension Tanimoto chemical similarity score between small molecules was calculated by the SMSD software.

### Survival analysis of breast cancer patients

In this study, we identified 19 potential small molecule drugs for breast cancer, in which 12 drugs have been validated by previous publication. Next, we further investigated whether the miRNAs that associated with the potential breast cancer drugs could distinguish breast cancer patients with good or poor outcome. Thus, we first collected breast cancer samples with miRNA expression and clinical information from TCGA and GEO database (GSE19783). As a result, we obtained 465 and 99 breast cancer patients from these two data resources, respectively. Then, we used the *K*-means cluster method (*K*=2) to cluster the patients into two groups based on miRNA expression. Finally, we used log-rank test to evaluate the statistical significant of difference between the two groups, and drew the Kaplan-Meier curve of the two different prognostic groups with R package *survival*.

## SUPPLEMENTARY FIGURES AND TABLES






