# Supplementary material for: Identification of associations between small molecule drugs and miRNAs based on functional similarity
Source: Oncotarget. 2016 May 24;7(25):38658–69. doi: 10.18632/oncotarget.9577 (PMC5122418; doi:10.18632/oncotarget.9577)
Supplement: Supplementary file 1 [file oncotarget-07-38658-s001.pdf]

Identification of associations between small molecule drugs and miRNAs based on functional similarity

SUPPLEMENTARY FIGURES AND TABLES

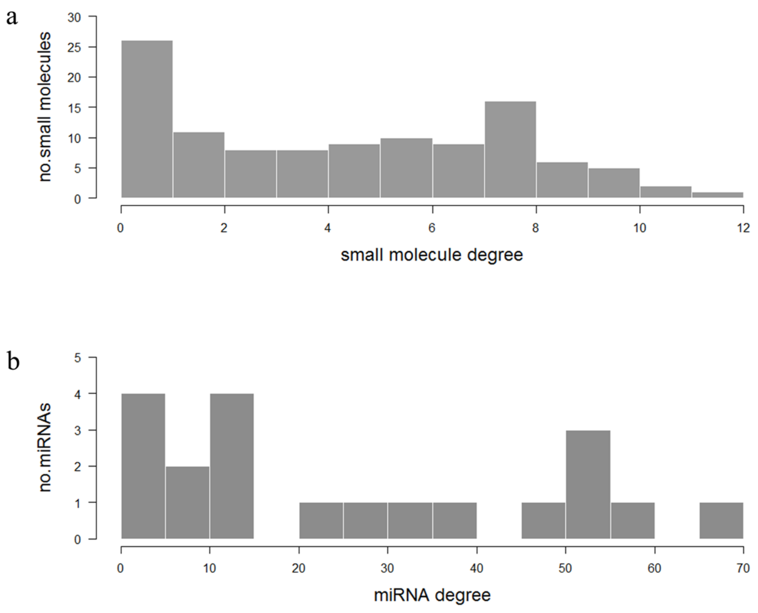

Supplementary Figure S1: The degree distribution of small molecules and miRNAs. As shown in **a.** most small molecules (~40%) connected a small number of miRNAs (less than 20%). In **b.** most miRNAs (50%) connected with a small number of small molecules (less than 20%).

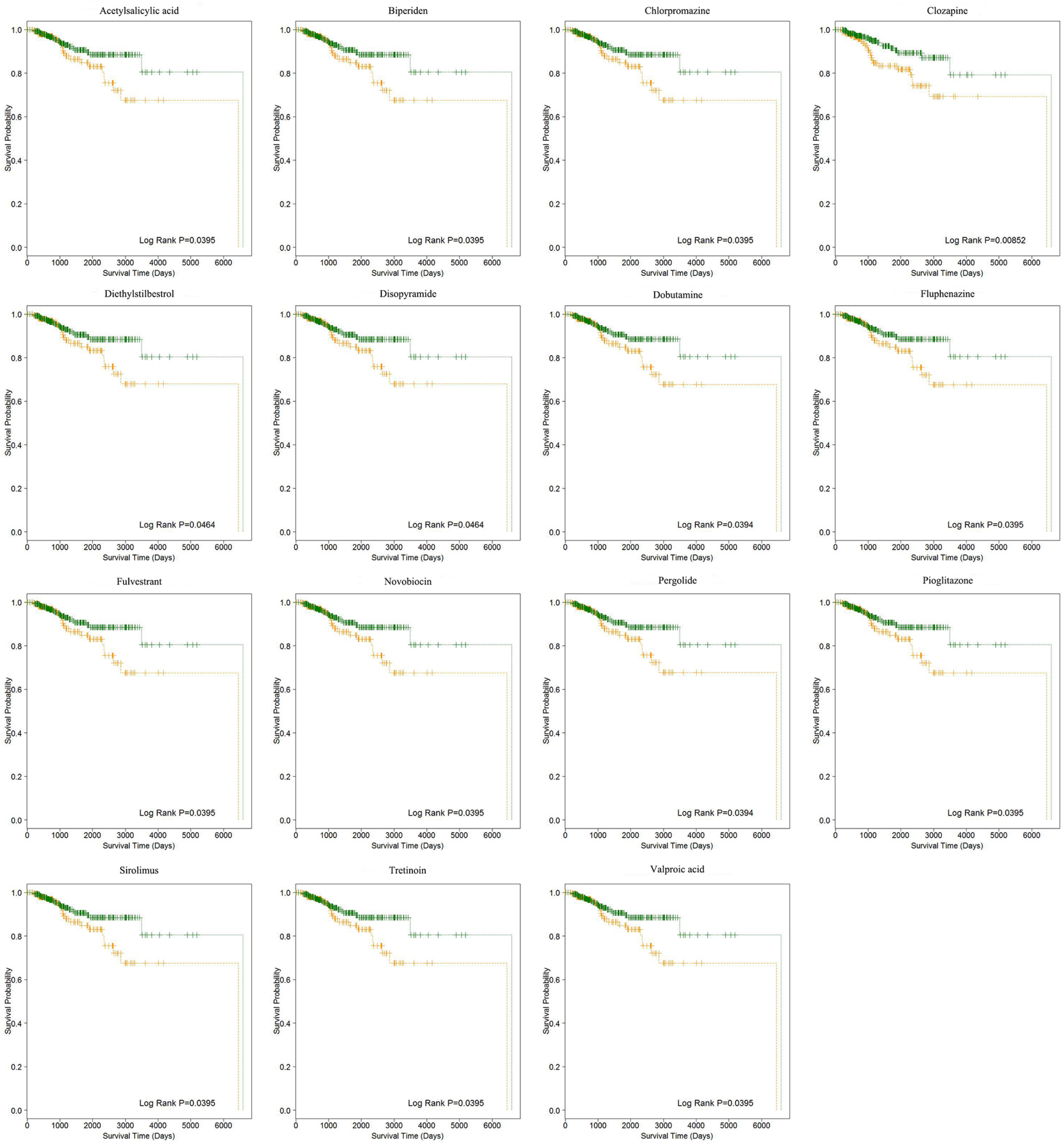

Supplementary Figure S2: The survival analysis of breast cancer patients in the TCGA dataset. 15 small molecule-related miRNAs could classify the breast cancer patients into high-risk and low-risk groups significantly in TCGA dataset.

Supplementary Table S1: The detailed information for predicted associations. Column MiRNA indicated the miRNA of perturbation. Column Small molecule represented the name of small molecule. Column Score reflected the degree of functional similarity between the miRNA and the small molecule.

See Supplementary File S1

Supplementary Table S2: Details of miRNA-perturbed data. The table provided the detail information for data of miRNA perturbation. Abbreviations, first column: MiRNA, transfected miRNA. Second column, GSE, the accession number of GEO. Third column, Mimics/Inhibitors, the miRNA mimics or inhibitors. Fourth column, Conditions, miRNA transfected conditions(eg: the cell or cell lines). Last column, GPL, the platform that one set of data used.

See Supplementary File S2
