# Supplementary material for: Identification of associations between small molecule drugs and miRNAs based on functional similarity
Source: Oncotarget. 2016 May 24;7(25):38658–69. doi: 10.18632/oncotarget.9577 (PMC5122418; doi:10.18632/oncotarget.9577)
Supplement: Supplementary file 2 [file oncotarget-07-38658-s002.docx]

Table S1. The detailed information for predicted associations. Column MiRNA indicated the miRNA of perturbation. Column Small molecule represented the name of small molecule. Column Score reflected the degree of functional similarity between the miRNA and the small molecule.

| MiRNA | Small molecule | Score |
| --- | --- | --- |
| miR-21 | terconazole | 1 |
| miR-26a | terconazole | 1 |
| miR-30e* | terconazole | 1 |
| miR-373 | bromopride | 0.821 |
| miR-26a | thioridazine | 0.815 |
| miR-26a | fulvestrant | 0.811 |
| miR-26a | LY-294002 | 0.806 |
| miR-26a | sirolimus | 0.806 |
| miR-493 | bromopride | 0.805 |
| miR-21 | valproic acid | 0.804 |
| miR-493 | benfotiamine | 0.8 |
| miR-26a | geldanamycin | 0.799 |
| miR-145 | LY-294002 | 0.798 |
| miR-200c | pergolide | 0.798 |
| miR-30e* | sirolimus | 0.797 |
| miR-145 | sirolimus | 0.796 |
| miR-26a | monorden | 0.796 |
| miR-200c | bromopride | 0.794 |
| miR-21 | dobutamine | 0.794 |
| miR-200c | morantel | 0.793 |
| miR-26a | DL-PPMP | 0.793 |
| miR-26a | estradiol | 0.792 |
| miR-26a | wortmannin | 0.792 |
| miR-30e* | LY-294002 | 0.792 |
| miR-26a | fluphenazine | 0.791 |
| miR-26a | CP-645525-01 | 0.791 |
| miR-10a | bromopride | 0.79 |
| miR-26a | haloperidol | 0.79 |
| miR-373 | ionomycin | 0.79 |
| miR-21 | chlorpromazine | 0.789 |
| miR-145 | wortmannin | 0.788 |
| miR-100 | bromopride | 0.787 |
| miR-145 | DL-PPMP | 0.787 |
| miR-30e* | wortmannin | 0.787 |
| miR-200c | celastrol | 0.786 |
| miR-200c | ionomycin | 0.786 |
| miR-26a | alpha-estradiol | 0.786 |
| miR-493 | ionomycin | 0.786 |
| miR-21 | fluphenazine | 0.785 |
| miR-26a | tanespimycin | 0.784 |
| miR-145 | thioridazine | 0.783 |
| miR-133a | geldanamycin | 0.782 |
| miR-145 | estradiol | 0.782 |
| miR-335 | thioridazine | 0.782 |
| miR-335 | wortmannin | 0.782 |
| miR-335 | geldanamycin | 0.781 |
| miR-30e* | haloperidol | 0.78 |
| miR-335 | LY-294002 | 0.78 |
| miR-493 | pergolide | 0.78 |
| miR-21 | pergolide | 0.779 |
| miR-30e* | estradiol | 0.779 |
| miR-373 | pergolide | 0.779 |
| miR-30e* | thioridazine | 0.778 |
| miR-145 | fulvestrant | 0.777 |
| miR-21 | geldanamycin | 0.777 |
| miR-26a | novobiocin | 0.777 |
| miR-30e* | monorden | 0.777 |
| miR-335 | tanespimycin | 0.777 |
| miR-133a | tanespimycin | 0.776 |
| miR-145 | haloperidol | 0.776 |
| miR-200c | valproic acid | 0.776 |
| miR-200c | disopyramide | 0.776 |
| miR-21 | 5186223 | 0.776 |
| miR-26a | naltrexone | 0.776 |
| miR-21 | disopyramide | 0.775 |
| miR-30e* | fulvestrant | 0.775 |
| miR-200c | sulindac sulfide | 0.774 |
| miR-30e* | geldanamycin | 0.774 |
| miR-200c | resveratrol | 0.773 |
| miR-21 | foliosidine | 0.773 |
| miR-133a | valproic acid | 0.772 |
| miR-133a | foliosidine | 0.771 |
| miR-145 | alpha-estradiol | 0.771 |
| miR-200c | chlorpromazine | 0.771 |
| miR-200c | dobutamine | 0.771 |
| miR-373 | morantel | 0.771 |
| miR-26a | valproic acid | 0.77 |
| miR-26a | acetylsalicylic acid | 0.77 |
| miR-100 | ionomycin | 0.769 |
| miR-133a | thioridazine | 0.769 |
| miR-133a | DL-PPMP | 0.769 |
| miR-335 | biperiden | 0.769 |
| miR-21 | celastrol | 0.768 |
| miR-21 | CP-645525-01 | 0.768 |
| miR-26a | 7-aminocephalosporanic acid | 0.768 |
| miR-335 | 7-aminocephalosporanic acid | 0.768 |
| miR-373 | benfotiamine | 0.768 |
| miR-21 | tanespimycin | 0.767 |
| miR-21 | morantel | 0.767 |
| miR-21 | 5666823 | 0.766 |
| miR-21 | 15-delta prostaglandin J2 | 0.766 |
| miR-30e* | acetylsalicylic acid | 0.766 |
| miR-335 | estradiol | 0.766 |
| miR-373 | sulindac sulfide | 0.766 |
| miR-100 | pergolide | 0.765 |
| miR-21 | ionomycin | 0.765 |
| miR-335 | acetylsalicylic acid | 0.765 |
| miR-373 | celastrol | 0.765 |
| miR-373 | dobutamine | 0.765 |
| miR-26a | chlorpromazine | 0.764 |
| miR-26a | troglitazone | 0.764 |
| miR-30e* | alpha-estradiol | 0.764 |
| miR-493 | resveratrol | 0.764 |
| miR-133a | chlorpromazine | 0.763 |
| miR-133a | estradiol | 0.763 |
| miR-26a | santonin | 0.763 |
| miR-26a | suxibuzone | 0.763 |
| miR-335 | valproic acid | 0.763 |
| miR-133a | wortmannin | 0.762 |
| miR-21 | glycocholic acid | 0.762 |
| miR-30e* | DL-PPMP | 0.762 |
| miR-335 | monorden | 0.762 |
| miR-200c | 5186223 | 0.761 |
| miR-21 | bromopride | 0.761 |
| miR-21 | dexverapamil | 0.761 |
| miR-10a | ionomycin | 0.76 |
| miR-26a | vorinostat | 0.76 |
| miR-26a | nordihydroguaiaretic acid | 0.76 |
| miR-493 | dexverapamil | 0.76 |
| miR-100 | morantel | 0.759 |
| miR-145 | monorden | 0.759 |
| miR-145 | geldanamycin | 0.759 |
| miR-133a | LY-294002 | 0.758 |
| miR-335 | alpha-estradiol | 0.758 |
| miR-335 | foliosidine | 0.758 |
| miR-145 | tanespimycin | 0.757 |
| miR-21 | LY-294002 | 0.757 |
| miR-30e* | fluphenazine | 0.757 |
| miR-181 | biperiden | 0.756 |
| miR-200c | dexverapamil | 0.756 |
| miR-26a | phentolamine | 0.756 |
| miR-101 | acetylsalicylsalicylic acid | 0.755 |
| miR-145 | phentolamine | 0.755 |
| miR-21 | sulindac sulfide | 0.755 |
| miR-21 | vorinostat | 0.755 |
| miR-30e* | tanespimycin | 0.755 |
| miR-30e* | novobiocin | 0.755 |
| miR-335 | 15-delta prostaglandin J2 | 0.755 |
| miR-373 | disopyramide | 0.755 |
| miR-100 | dexverapamil | 0.754 |
| miR-200c | tanespimycin | 0.754 |
| miR-21 | equilin | 0.754 |
| miR-26a | 15-delta prostaglandin J2 | 0.754 |
| miR-26a | etofylline | 0.754 |
| miR-30e* | nordihydroguaiaretic acid | 0.754 |
| miR-30e* | phentolamine | 0.754 |
| miR-335 | nordihydroguaiaretic acid | 0.754 |
| miR-100 | benfotiamine | 0.753 |
| miR-10a | dexverapamil | 0.753 |
| miR-200c | geldanamycin | 0.753 |
| miR-26a | hydralazine | 0.753 |
| miR-373 | valproic acid | 0.753 |
| miR-100 | celastrol | 0.752 |
| miR-145 | nordihydroguaiaretic acid | 0.752 |
| miR-145 | CP-645525-01 | 0.752 |
| miR-21 | resveratrol | 0.752 |
| miR-21 | AG-013608 | 0.752 |
| miR-26a | pioglitazone | 0.752 |
| miR-26a | azathioprine | 0.752 |
| miR-30e* | N-phenylanthranilic acid | 0.752 |
| miR-335 | sirolimus | 0.752 |
| miR-373 | resveratrol | 0.752 |
| miR-100 | dobutamine | 0.751 |
| miR-133a | 7-aminocephalosporanic acid | 0.751 |
| miR-145 | fluphenazine | 0.751 |
| miR-200c | tretinoin | 0.751 |
| miR-21 | fulvestrant | 0.751 |
| miR-21 | estradiol | 0.751 |
| miR-21 | thioridazine | 0.751 |
| miR-373 | dexverapamil | 0.751 |
| miR-133a | monorden | 0.75 |
| miR-145 | troglitazone | 0.75 |
| miR-145 | acetylsalicylic acid | 0.75 |
| miR-145 | N-phenylanthranilic acid | 0.75 |
| miR-21 | pioglitazone | 0.75 |
| miR-26a | biperiden | 0.75 |
| miR-30e* | 15-delta prostaglandin J2 | 0.75 |
| miR-335 | chlorpromazine | 0.75 |
| miR-133a | fulvestrant | 0.749 |
| miR-200c | fluphenazine | 0.749 |
| miR-200c | foliosidine | 0.749 |
| miR-21 | monorden | 0.749 |
| miR-10a | pergolide | 0.748 |
| miR-133a | fluphenazine | 0.748 |
| miR-133a | acetylsalicylic acid | 0.748 |
| miR-200c | monorden | 0.748 |
| miR-200c | benfotiamine | 0.748 |
| miR-21 | acetylsalicylic acid | 0.748 |
| miR-145 | hydralazine | 0.747 |
| miR-26a | prochlorperazine | 0.747 |
| miR-30e* | 7-aminocephalosporanic acid | 0.747 |
| miR-335 | pepstatin | 0.747 |
| miR-493 | sulindac sulfide | 0.747 |
| miR-101 | azapropazone | 0.746 |
| miR-133a | alpha-estradiol | 0.746 |
| miR-145 | novobiocin | 0.746 |
| miR-26a | tretinoin | 0.746 |
| miR-335 | DL-PPMP | 0.746 |
| miR-133a | sirolimus | 0.745 |
| miR-133a | celastrol | 0.745 |
| miR-21 | tretinoin | 0.745 |
| miR-30e* | pioglitazone | 0.745 |
| miR-335 | haloperidol | 0.745 |
| miR-335 | fluphenazine | 0.745 |
| miR-493 | celastrol | 0.745 |
| miR-145 | 7-aminocephalosporanic acid | 0.744 |
| miR-145 | suxibuzone | 0.744 |
| miR-133a | disopyramide | 0.743 |
| miR-145 | etofylline | 0.743 |
| miR-21 | biperiden | 0.743 |
| miR-26a | meclofenamic acid | 0.743 |
| miR-30e* | vorinostat | 0.743 |
| miR-10a | benfotiamine | 0.742 |
| miR-133a | biperiden | 0.742 |
| miR-145 | vorinostat | 0.742 |
| miR-200c | 5666823 | 0.742 |
| miR-200c | pioglitazone | 0.742 |
| miR-200c | dexamethasone | 0.742 |
| miR-200c | amoxapine | 0.742 |
| miR-30e* | hydralazine | 0.742 |
| miR-493 | dobutamine | 0.742 |
| miR-145 | pioglitazone | 0.741 |
| miR-181 | metaraminol | 0.741 |
| miR-26a | foliosidine | 0.741 |
| miR-26a | chloropyramine | 0.741 |
| miR-30e* | troglitazone | 0.741 |
| miR-335 | fulvestrant | 0.741 |
| miR-374a | valproic acid | 0.741 |
| miR-145 | naltrexone | 0.74 |
| miR-145 | PNU-0293363 | 0.74 |
| miR-21 | proscillaridin | 0.74 |
| miR-26a | gefitinib | 0.74 |
| miR-26a | naphazoline | 0.74 |
| miR-26a | PNU-0293363 | 0.74 |
| miR-374a | estradiol | 0.74 |
| miR-100 | resveratrol | 0.739 |
| miR-21 | naftidrofuryl | 0.739 |
| miR-30e* | valproic acid | 0.739 |
| miR-374a | fulvestrant | 0.739 |
| miR-10a | dobutamine | 0.738 |
| miR-10a | morantel | 0.738 |
| miR-133a | nordihydroguaiaretic acid | 0.738 |
| miR-145 | naphazoline | 0.738 |
| miR-181 | vorinostat | 0.738 |
| miR-26a | alvespimycin | 0.738 |
| miR-335 | pioglitazone | 0.738 |
| miR-335 | eldeline | 0.738 |
| miR-335 | meclofenamic acid | 0.738 |
| miR-10a | resveratrol | 0.737 |
| miR-145 | valproic acid | 0.737 |
| miR-21 | sulfaguanidine | 0.737 |
| miR-26a | methotrexate | 0.737 |
| miR-335 | proscillaridin | 0.737 |
| miR-335 | glycocholic acid | 0.737 |
| miR-373 | chlorpromazine | 0.737 |
| miR-373 | 5186223 | 0.737 |
| miR-145 | biperiden | 0.736 |
| miR-181 | valproic acid | 0.736 |
| miR-21 | etofylline | 0.736 |
| miR-21 | nordihydroguaiaretic acid | 0.736 |
| miR-26a | N-phenylanthranilic acid | 0.736 |
| miR-335 | suxibuzone | 0.736 |
| miR-374a | geldanamycin | 0.736 |
| miR-1 | wortmannin | 0.735 |
| miR-133a | nefopam | 0.735 |
| miR-145 | prochlorperazine | 0.735 |
| miR-26a | proscillaridin | 0.735 |
| miR-374a | wortmannin | 0.735 |
| miR-125b | sulfamethoxazole | 0.734 |
| miR-133a | CP-645525-01 | 0.734 |
| miR-181 | geldanamycin | 0.734 |
| miR-21 | oxaprozin | 0.734 |
| miR-30e* | naltrexone | 0.734 |
| miR-145 | methotrexate | 0.733 |
| miR-181 | foliosidine | 0.733 |
| miR-21 | metaraminol | 0.733 |
| miR-26a | clozapine | 0.733 |
| miR-26a | AG-013608 | 0.733 |
| miR-30e* | promazine | 0.733 |
| miR-30e* | meclofenamic acid | 0.733 |
| miR-373 | 5666823 | 0.733 |
| miR-374a | clozapine | 0.733 |
| miR-493 | 5186223 | 0.733 |
| miR-1 | LY-294002 | 0.732 |
| miR-10a | sulindac sulfide | 0.732 |
| miR-133a | tretinoin | 0.732 |
| miR-200c | vorinostat | 0.732 |
| miR-200c | CP-645525-01 | 0.732 |
| miR-374a | proscillaridin | 0.732 |
| miR-200c | estradiol | 0.731 |
| miR-200c | naftidrofuryl | 0.731 |
| miR-21 | alpha-estradiol | 0.731 |
| miR-21 | rosiglitazone | 0.731 |
| miR-21 | novobiocin | 0.731 |
| miR-21 | amoxapine | 0.731 |
| miR-21 | diethylstilbestrol | 0.731 |
| miR-335 | amoxapine | 0.731 |
| miR-1 | DL-PPMP | 0.73 |
| miR-133a | 15-delta prostaglandin J2 | 0.73 |
| miR-133a | vorinostat | 0.73 |
| miR-145 | alvespimycin | 0.73 |
| miR-145 | chloropyramine | 0.73 |
| miR-181 | nefopam | 0.73 |
| miR-200c | acetylsalicylic acid | 0.73 |
| miR-200c | rosiglitazone | 0.73 |
| miR-21 | dexamethasone | 0.73 |
| miR-29 | bromopride | 0.73 |
| miR-30e* | etofylline | 0.73 |
| miR-30e* | pepstatin | 0.73 |
| miR-335 | phentolamine | 0.73 |
| miR-374a | LY-294002 | 0.73 |
| miR-374a | monorden | 0.73 |
| miR-374a | pioglitazone | 0.73 |
| miR-10a | celastrol | 0.729 |
| miR-133a | amoxapine | 0.729 |
| miR-181 | glycocholic acid | 0.729 |
| miR-21 | sirolimus | 0.729 |
| miR-30e* | clozapine | 0.729 |
| miR-30e* | cyclizine | 0.729 |
| miR-30e* | azathioprine | 0.729 |
| miR-335 | nefopam | 0.729 |
| miR-374a | phentolamine | 0.729 |
| miR-129 | wortmannin | 0.728 |
| miR-129 | gefitinib | 0.728 |
| miR-145 | clozapine | 0.728 |
| miR-145 | gefitinib | 0.728 |
| miR-181 | DL-PPMP | 0.728 |
| miR-181 | diethylstilbestrol | 0.728 |
| miR-200c | LY-294002 | 0.728 |
| miR-21 | 7-aminocephalosporanic acid | 0.728 |
| miR-30e* | chlorpromazine | 0.728 |
| miR-30e* | methotrexate | 0.728 |
| miR-30e* | biperiden | 0.728 |
| miR-335 | AG-013608 | 0.728 |
| miR-374a | haloperidol | 0.728 |
| miR-374a | biperiden | 0.728 |
| miR-145 | azathioprine | 0.727 |
| miR-181 | monorden | 0.727 |
| miR-181 | tretinoin | 0.727 |
| miR-200c | equilin | 0.727 |
| miR-200c | glycocholic acid | 0.727 |
| miR-26a | eldeline | 0.727 |
| miR-30e* | tretinoin | 0.727 |
| miR-335 | clozapine | 0.727 |
| miR-335 | disopyramide | 0.727 |
| miR-133a | haloperidol | 0.726 |
| miR-133a | glycocholic acid | 0.726 |
| miR-200c | AG-013608 | 0.726 |
| miR-21 | santonin | 0.726 |
| miR-26a | metaraminol | 0.726 |
| miR-30e* | proscillaridin | 0.726 |
| miR-335 | azathioprine | 0.726 |
| miR-374a | vorinostat | 0.726 |
| miR-374a | 7-aminocephalosporanic acid | 0.726 |
| miR-493 | morantel | 0.726 |
| miR-1 | tanespimycin | 0.725 |
| miR-1 | sirolimus | 0.725 |
| miR-125b | piperacetazine | 0.725 |
| miR-145 | 15-delta prostaglandin J2 | 0.725 |
| miR-181 | LY-294002 | 0.725 |
| miR-181 | estradiol | 0.725 |
| miR-21 | thalidomide | 0.725 |
| miR-21 | suxibuzone | 0.725 |
| miR-26a | naftidrofuryl | 0.725 |
| miR-30e* | CP-645525-01 | 0.725 |
| miR-30e* | gefitinib | 0.725 |
| miR-335 | dexverapamil | 0.725 |
| miR-335 | monastrol | 0.725 |
| miR-374a | hydroxyzine | 0.725 |
| miR-1 | estradiol | 0.724 |
| miR-133a | pioglitazone | 0.724 |
| miR-145 | AG-013608 | 0.724 |
| miR-181 | fluphenazine | 0.724 |
| miR-181 | meclofenamic acid | 0.724 |
| miR-21 | troglitazone | 0.724 |
| miR-21 | naltrexone | 0.724 |
| miR-26a | PNU-0230031 | 0.724 |
| miR-30e* | alvespimycin | 0.724 |
| miR-335 | troglitazone | 0.724 |
| miR-335 | vorinostat | 0.724 |
| miR-335 | etofylline | 0.724 |
| miR-335 | novobiocin | 0.724 |
| miR-335 | propantheline bromide | 0.724 |
| miR-335 | gefitinib | 0.724 |
| miR-335 | PNU-0293363 | 0.724 |
| miR-100 | disopyramide | 0.723 |
| miR-10a | 5666823 | 0.723 |
| miR-145 | (+)-chelidonine | 0.723 |
| miR-181 | thioridazine | 0.723 |
| miR-21 | PNU-0230031 | 0.723 |
| miR-26a | propantheline bromide | 0.723 |
| miR-335 | methotrexate | 0.723 |
| miR-100 | sulindac sulfide | 0.722 |
| miR-10a | 5186223 | 0.722 |
| miR-133a | rosiglitazone | 0.722 |
| miR-145 | parthenolide | 0.722 |
| miR-181 | 7-aminocephalosporanic acid | 0.722 |
| miR-200c | 15-delta prostaglandin J2 | 0.722 |
| miR-200c | F0447-0125 | 0.722 |
| miR-21 | C-75 | 0.722 |
| miR-21 | dopamine | 0.722 |
| miR-22 | wortmannin | 0.722 |
| miR-30e* | 0317956-0000 | 0.722 |
| miR-373 | dexamethasone | 0.722 |
| miR-374a | equilin | 0.722 |
| miR-129 | DL-PPMP | 0.721 |
| miR-133a | proscillaridin | 0.721 |
| miR-133a | meclofenamic acid | 0.721 |
| miR-145 | tretinoin | 0.721 |
| miR-181 | sirolimus | 0.721 |
| miR-181 | nordihydroguaiaretic acid | 0.721 |
| miR-21 | eldeline | 0.721 |
| miR-21 | Prestwick-864 | 0.721 |
| miR-26a | diethylstilbestrol | 0.721 |
| miR-26b | equilin | 0.721 |
| miR-335 | rosiglitazone | 0.721 |
| miR-335 | N-phenylanthranilic acid | 0.721 |
| miR-181 | disopyramide | 0.72 |
| miR-200c | novobiocin | 0.72 |
| miR-21 | wortmannin | 0.72 |
| miR-21 | beta-escin | 0.72 |
| miR-21 | monastrol | 0.72 |
| miR-26a | trifluoperazine | 0.72 |
| miR-30e* | bendroflumethiazide | 0.72 |
| miR-335 | naftidrofuryl | 0.72 |
| miR-335 | alvespimycin | 0.72 |
| miR-335 | santonin | 0.72 |
| miR-374a | isoetarine | 0.72 |
| miR-374a | eldeline | 0.72 |
| miR-374a | glycocholic acid | 0.72 |
| miR-493 | 5666823 | 0.72 |
| miR-181 | dexverapamil | 0.719 |
| miR-200c | trichostatin A | 0.719 |
| miR-200c | biperiden | 0.719 |
| miR-21 | haloperidol | 0.719 |
| miR-22 | monorden | 0.719 |
| miR-26b | valproic acid | 0.719 |
| miR-26b | promethazine | 0.719 |
| miR-30e* | propantheline bromide | 0.719 |
| miR-30e* | quipazine | 0.719 |
| miR-335 | bendroflumethiazide | 0.719 |
| miR-374a | DL-PPMP | 0.719 |
| miR-374a | lisuride | 0.719 |
| miR-493 | cefadroxil | 0.719 |
| miR-493 | dexpanthenol | 0.719 |
| miR-1 | thioridazine | 0.718 |
| miR-100 | 5186223 | 0.718 |
| miR-129 | estradiol | 0.718 |
| miR-133a | suxibuzone | 0.718 |
| miR-181 | acetylsalicylic acid | 0.718 |
| miR-181 | suxibuzone | 0.718 |
| miR-26a | celecoxib | 0.718 |
| miR-30e* | PNU-0230031 | 0.718 |
| miR-373 | naftidrofuryl | 0.718 |
| miR-373 | amoxapine | 0.718 |
| miR-374a | oxaprozin | 0.718 |
| miR-374a | hydralazine | 0.718 |
| miR-1 | phentolamine | 0.717 |
| miR-133a | dobutamine | 0.717 |
| miR-145 | proscillaridin | 0.717 |
| miR-145 | meclofenamic acid | 0.717 |
| miR-145 | santonin | 0.717 |
| miR-181 | rosiglitazone | 0.717 |
| miR-181 | phentolamine | 0.717 |
| miR-200c | alpha-estradiol | 0.717 |
| miR-21 | propantheline bromide | 0.717 |
| miR-22 | troglitazone | 0.717 |
| miR-22 | clozapine | 0.717 |
| miR-26a | morantel | 0.717 |
| miR-26a | benzonatate | 0.717 |
| miR-26a | amoxapine | 0.717 |
| miR-26a | pepstatin | 0.717 |
| miR-30e* | foliosidine | 0.717 |
| miR-30e* | suxibuzone | 0.717 |
| miR-335 | naltrexone | 0.717 |
| miR-374a | sirolimus | 0.717 |
| miR-1 | haloperidol | 0.716 |
| miR-1 | troglitazone | 0.716 |
| miR-1 | hydralazine | 0.716 |
| miR-100 | 5666823 | 0.716 |
| miR-125b | nitrendipine | 0.716 |
| miR-129 | vorinostat | 0.716 |
| miR-181 | mafenide | 0.716 |
| miR-181 | furaltadone | 0.716 |
| miR-200c | oxaprozin | 0.716 |
| miR-26b | proscillaridin | 0.716 |
| miR-30e* | prochlorperazine | 0.716 |
| miR-30e* | dopamine | 0.716 |
| miR-374a | CP-645525-01 | 0.716 |
| miR-129 | geldanamycin | 0.715 |
| miR-129 | lisuride | 0.715 |
| miR-133a | etofylline | 0.715 |
| miR-133a | sulfaguanidine | 0.715 |
| miR-145 | eldeline | 0.715 |
| miR-145 | diethylstilbestrol | 0.715 |
| miR-181 | 15-delta prostaglandin J2 | 0.715 |
| miR-181 | dexamethasone | 0.715 |
| miR-200c | cefadroxil | 0.715 |
| miR-21 | mafenide | 0.715 |
| miR-21 | ambroxol | 0.715 |
| miR-21 | azathioprine | 0.715 |
| miR-21 | PNU-0293363 | 0.715 |
| miR-26b | glycocholic acid | 0.715 |
| miR-373 | foliosidine | 0.715 |
| miR-373 | cefadroxil | 0.715 |
| miR-374a | troglitazone | 0.715 |
| miR-374a | etofylline | 0.715 |
| miR-374a | pepstatin | 0.715 |
| miR-1 | nordihydroguaiaretic acid | 0.714 |
| miR-129 | monorden | 0.714 |
| miR-145 | chlorpromazine | 0.714 |
| miR-145 | lanatoside C | 0.714 |
| miR-145 | promazine | 0.714 |
| miR-200c | thioridazine | 0.714 |
| miR-200c | suxibuzone | 0.714 |
| miR-30e* | parthenolide | 0.714 |
| miR-335 | tretinoin | 0.714 |
| miR-335 | CP-645525-01 | 0.714 |
| miR-335 | sulfaguanidine | 0.714 |
| miR-373 | butyl hydroxybenzoate | 0.714 |
| miR-100 | valproic acid | 0.713 |
| miR-133a | naftidrofuryl | 0.713 |
| miR-133a | santonin | 0.713 |
| miR-145 | quipazine | 0.713 |
| miR-181 | fulvestrant | 0.713 |
| miR-181 | etofylline | 0.713 |
| miR-181 | novobiocin | 0.713 |
| miR-181 | amoxapine | 0.713 |
| miR-181 | norfloxacin | 0.713 |
| miR-200c | sirolimus | 0.713 |
| miR-200c | C-75 | 0.713 |
| miR-26b | foliosidine | 0.713 |
| miR-26b | 7-aminocephalosporanic acid | 0.713 |
| miR-30e* | santonin | 0.713 |
| miR-30e* | PNU-0293363 | 0.713 |
| miR-373 | tanespimycin | 0.713 |
| miR-374a | pirinixic acid | 0.713 |
| miR-493 | disopyramide | 0.713 |
