# Supplementary material for: Identification of associations between small molecule drugs and miRNAs based on functional similarity
Source: Oncotarget. 2016 May 24;7(25):38658–69. doi: 10.18632/oncotarget.9577 (PMC5122418; doi:10.18632/oncotarget.9577)
Supplement: Supplementary file 3 [file oncotarget-07-38658-s003.docx]

Table S2. Details of miRNA-perturbed data. The table provided the detail information for data of miRNA perturbation. Abbreviations, first column: MiRNA, transfected miRNA. Second column, GSE, the accession number of GEO. Third column, Mimics/Inhibitors, the miRNA mimics or inhibitors. Fourth column, Conditions, miRNA transfected conditions(eg: the cell or cell lines) . Last column, GPL, the platform that one set of data used.

| **MiRNA** | **GSE** | **Mimics/Inhibitors** | **Conditions** | **GPL** |
| --- | --- | --- | --- | --- |
| miR-98 | GSE10292 | M | Hela | GPL571 |
| miR-34b | GSE10455 | M | HCT116 | GPL4133 |
| miR-34c | GSE10455 | M | HCT116 | GPL4133 |
| miR-155 | GSE10864 | M | BuRkitt’s Lymphoma | GPL5770 |
| miR-205 | GSE11701 | M | DU145 | GPL6104 |
| miR-1 | GSE11968 | M | HeLa | GPL4133 |
| miR-124 | GSE11968 | M | HeLa | GPL4133 |
| miR-181 | GSE11968 | M | HeLa | GPL4133 |
| miR-26b | GSE12091 | M | HeLa | GPL571 |
| let-7f | GSE12615 | M | UCI101 | GPL6104 |
| let-7f | GSE12615 | M | BG1 | GPL6104 |
| miR-200c | GSE12615 | M | BG1 | GPL6104 |
| miR-424 | GSE12615 | M | BG1 | GPL6104 |
| miR-98 | GSE12615 | M | BG1 | GPL6104 |
| miR-155 | GSE13296 | I | DendRitic Cells (DC)+LPS | GPL570 |
| miR-155 | GSE13296 | I | DendRitic Cells (DC) | GPL570 |
| miR-101 | GSE13674 | M | UM-UC-3 | GPL5799 |
| miR-7 | GSE14507 | M | A549 | GPL570 |
| miR-124 | GSE14537 | M | FLPin HEK 293 | GPL570 |
| miR-7 | GSE14537 | M | FLPin HEK 293 | GPL570 |
| miR-106a | GSE14831 | M | HCT116 DiceR-/- #2 | GPL4372 |
| miR-106b | GSE14831 | M | HCT116 DiceR-/- #2 | GPL4372 |
| miR-17-3p | GSE14831 | M | HCT116 DiceR-/- #2 | GPL4372 |
| miR-17-5p | GSE14831 | M | HCT116 DiceR-/- #2 | GPL4372 |
| miR-20 | GSE14831 | M | HCT116 DiceR-/- #2 | GPL4372 |
| miR-18a | GSE14847 | M | MCF7 | GPL6102 |
| miR-196a | GSE15576 | M | MCF7 | GPL4133 |
| miR-9 | GSE15749 | M | MCF7 | GPL570 |
| miR-9* | GSE15749 | M | MCF7 | GPL570 |
| miR-181c | GSE16239 | M | KATO-III | GPL6480 |
| miR-22 | GSE16568 | M | ES2 | GPL6947 |
| miR-30a | GSE16569 | I | OVSAYO | GPL6947 |
| miR-100 | GSE16571 | M | OVSAYO | GPL6947 |
| mir-182 | GSE16572 | I | ES2 | GPL6947 |
| miR-34a | GSE16674 | M | K562 | GPL570 |
| miR-31 | GSE16700 | M | OVCAR8 | GPL6947 |
| miR-31 | GSE16908 | M | LEC | GPL2986 |
| miR-210 | GSE16962 | M | HUVEC | GPL570 |
| miR-210 | GSE16962 | I | HUVEC | GPL570 |
| miR-130b | GSE17386 | M | CD133- HCC | GPL570 |
| miR-26a | GSE17460 | M | MCF7 | GPL5175 |
| miR-24 | GSE17828 | M | HepG2 | GPL5104 |
| miR-193b | GSE18510 | M | Malme-3M | GPL6480 |
| miR-145 | GSE18625 | M | DLD1 | GPL570 |
| miR-29 | GSE18651 | M | IMR-90 | GPL6244 |
| miR-210 | GSE18695 | M | A549 | GPL1456 |
| miR-34a | GSE18695 | M | A549 | GPL1456 |
| miR-124 | GSE18835 | M | HEK293T | GPL9494 |
| miR-124 | GSE18835 | M | HEK293T | GPL9497 |
| miR-335 | GSE19232 | M | hMSC | GPL6480 |
| miR-125b | GSE19680 | M | CMK | GPL570 |
| miR-20a | GSE19688 | M | Hs683 | GPL570 |
| miR-133a | GSE19717 | M | KK47 | GPL4133 |
| miR-133a | GSE19717 | M | T24 | GPL4133 |
| miR-145 | GSE19717 | M | KK47 | GPL4133 |
| miR-145 | GSE19717 | M | T24 | GPL4133 |
| miR-489 | GSE19718 | M | FaDu | GPL4133 |
| miR-489 | GSE19718 | M | HSC3 | GPL4133 |
| miR-504 | GSE19718 | M | FaDu | GPL4133 |
| miR-504 | GSE19718 | M | HSC3 | GPL4133 |
| miR-221 | GSE19777 | I | MCF7-FR | GPL570 |
| miR-222 | GSE19777 | I | MCF7-FR | GPL570 |
| miR-483 | GSE19931 | M | HaCaT | GPL1456 |
| miR-129 | GSE19933 | M | TCP1 | GPL1456 |
| miR-133a | GSE20028 | M | SAS | GPL4133 |
| miR-133a | GSE20028 | M | HSC3 | GPL4133 |
| miR-145 | GSE20028 | M | TE13 | GPL4133 |
| miR-145 | GSE20028 | M | TE2 | GPL4133 |
| miR-30e* | GSE20293 | M | SNB19 | GPL6848 |
| miR-30e* | GSE20293 | M | U-87MG | GPL6848 |
| miR-100 | GSE20668 | M | endothelials cells | GPL6480 |
| miR-517a | GSE20679 | I | Human hepatocellulaR caRcinoma cell line | GPL6244 |
| miR-663 | GSE20739 | I | HUVEC | GPL6947 |
| miR-17 | GSE20745 | I | HUVEC | GPL570 |
| miR-125b | GSE21458 | M | HUVEC | GPL6883 |
| miR-34a | GSE21832 | M | MDAMB231/Qmimic | GPL570 |
| miR-34a | GSE21832 | M | MDAMB231/Dmimic | GPL570 |
| miR-449b | GSE22143 | M | HAEC | GPL6244 |
| miR-1 | GSE24782 | M | BOY | GPL4133 |
| miR-1 | GSE24782 | M | T24 | GPL4133 |
| miR-1 | GSE24782 | M | A498 | GPL10332 |
| miR-1 | GSE24782 | M | HSC3 | GPL10332 |
| miR-1 | GSE24782 | M | FaDu | GPL10332 |
| miR-145 | GSE24782 | M | PC3 | GPL10332 |
| miR-145 | GSE24782 | M | DU145 | GPL10332 |
| miR-218 | GSE24782 | M | BOY | GPL10332 |
| miR-218 | GSE24782 | M | T24 | GPL4133 |
| miR-517a | GSE24782 | M | BOY | GPL4133 |
| miR-517a | GSE24782 | M | T24 | GPL4133 |
| miR-874 | GSE24782 | M | IMC3 | GPL10332 |
| miR-874 | GSE24782 | M | SAS | GPL10332 |
| miR-145 | GSE24980 | M | HCE | GPL6480 |
| miR-193b | GSE25215 | M | MIA PaCa-2 | GPL6480 |
| miR-124 | GSE25224 | M | HCT116 | GPL10379 |
| miR-1 | GSE26032 | M | IMC3 | GPL10332 |
| miR-1 | GSE26032 | M | DU145 | GPL10332 |
| miR-1 | GSE26032 | M | PC3 | GPL10332 |
| miR-133a | GSE26032 | M | H157 | GPL10332 |
| miR-133a | GSE26032 | M | P10 | GPL10332 |
| miR-133a | GSE26032 | M | A498 | GPL10332 |
| miR-133a | GSE26032 | M | IMC3 | GPL10332 |
| miR-133a | GSE26032 | M | DU145 | GPL10332 |
| miR-133a | GSE26032 | M | PC3 | GPL10332 |
| miR-135a | GSE26032 | M | A498 | GPL10332 |
| miR-183 | GSE26032 | M | KK47 | GPL10332 |
| miR-183 | GSE26032 | M | T24 | GPL10332 |
| miR-375 | GSE26032 | M | SAS | GPL10332 |
| miR-375 | GSE26032 | M | FaDu | GPL10332 |
| miR-96 | GSE26032 | M | T24 | GPL10332 |
| miR-96 | GSE26032 | M | KK47 | GPL10332 |
| miR-99a | GSE26332 | M | C4-2 | GPL570 |
| miR-184 | GSE26545 | M | SH-SY5Y | GPL6879 |
| miR-184 | GSE26545 | M | SK-N-SH | GPL6879 |
| miR-299 | GSE26545 | M | SH-SY5Y | GPL6879 |
| miR-299 | GSE26545 | M | SK-N-SH | GPL6879 |
| miR-34c | GSE26545 | M | SH-SY5Y | GPL6879 |
| miR-34c | GSE26545 | M | SK-N-SH | GPL6879 |
| miR-383 | GSE26545 | M | SH-SY5Y | GPL6879 |
| miR-383 | GSE26545 | M | SK-N-SH | GPL6879 |
| miR-487a | GSE26545 | M | SH-SY5Y | GPL6879 |
| miR-487a | GSE26545 | M | SK-N-SH | GPL6879 |
| miR-128 | GSE27431 | M | HEY/MAS5 | GPL570 |
| miR-128 | GSE27431 | M | HEY/plier | GPL570 |
| miR-128 | GSE27431 | M | HEY/GCRMA | GPL570 |
| miR-7 | GSE27431 | M | HEY/MAS5 | GPL570 |
| miR-7 | GSE27431 | M | HEY/piler | GPL570 |
| miR-7 | GSE27431 | M | HEY/GCRMA | GPL570 |
| miR-9 | GSE27529 | I | L428 | GPL570 |
| miR-204 | GSE28400 | M | HEK293 | GPL4133 |
| miR-142 | GSE28456 | M | Raji | GPL4133 |
| miR-1 | GSE28522 | M | HeLa | GPL4133 |
| miR-1 | GSE28522 | M | NPC-TWO1 | GPL4133 |
| miR-31 | GSE28810 | M | U251 | GPL570 |
| miR-10a | GSE29043 | M | HEK293T | GPL10558 |
| miR-10b | GSE29043 | M | HEK293T | GPL10558 |
| mir-30a | GSE29207 | M | hMADS cells, B9 | GPL13607 |
| mir-30a | GSE29207 | M | hMADS cells, B7 | GPL13607 |
| mir-30d | GSE29207 | M | hMADS cells, B9 | GPL13607 |
| miR-21 | GSE29242 | I | Siha cells | GPL8380 |
| miR-146b | GSE29496 | M | A549/vec cells | GPL6883 |
| miR-1 | GSE29760 | M | HCT116 | GPL4133 |
| miR-205 | GSE29782 | I | RWPE-1 | GPL6947 |
| miR-101 | GSE31397 | M | MCF7 | GPL570 |
| miR-1 | GSE31620 | M | LNCaP | GPL571 |
| miR-206 | GSE31620 | M | LNCaP | GPL571 |
| miR-27b | GSE31620 | M | LNCaP | GPL571 |
| miR-493 | GSE31751 | M | HCT116 | GPL13497 |
| miR-124 | GSE32876 | M | PDGFRA amplified neuRospheRe cell lines | GPL570 |
| miR-132 | GSE32876 | M | PDGFRA amplified neuRospheRe cell lines | GPL570 |
| miR-380 | GSE32876 | M | PDGFRA amplified neuRospheRe cell lines(Platelet-deRived gRowth factoR (PDGF)) | GPL570 |
| miR-433 | GSE32876 | M | PDGFRA amplified neuRospheRe cell lines | GPL570 |
| miR-448 | GSE32876 | M | PDGFRA amplified neuRospheRe cell lines | GPL570 |
| miR-520c | GSE32999 | M | PC3 | GPL6480 |
| miR-147a | GSE33247 | M | A549 | GPL1456 |
| miR-147b | GSE33247 | M | A549 | GPL1456 |
| miR-210 | GSE33247 | M | A549 | GPL1456 |
| miR-182 | GSE33293 | M | SNB19 | GPL6480 |
| miR-34c | GSE33337 | M | BEAS-2B | GPL6947 |
| miR-34c | GSE33337 | M | HFL1 | GPL6947 |
| miR-143 | GSE33420 | M | DLD1 | GPL570 |
| miR-365-2 | GSE33672 | M | NCI-H441 | GPL6244 |
| miR-9 | GSE33952 | M | epithelial breast cancer cell line | GPL6884 |
| miR-34a | GSE34242 | M | TS543 pRoneuRal GBM | GPL6947 |
| miR-106b | GSE34893 | M | LNCaP | GPL571 |
| miR-181b | GSE35030 | M | HUVECs | GPL1456 |
| miR-181a | GSE3605 | M | K562 | GPL885 |
| miR-1204 | GSE37185 | I | BT549 | GPL3921 |
| miR-1204 | GSE37185 | I | HEY | GPL3921 |
| miR-1204 | GSE37185 | I | MDAMB231 | GPL3921 |
| miR-1204 | GSE37185 | M | OV90 | GPL3921 |
| miR-1204 | GSE37185 | I | OVCAR8 | GPL3921 |
| miR-1204 | GSE37185 | M | SKBR3 | GPL3921 |
| miR-1204 | GSE37185 | I | SUM159PT | GPL3921 |
| miR-200c | GSE38817 | M | MSMC/502M | GPL10558 |
| miR-200c | GSE38817 | M | LSMC/502L | GPL10558 |
| miR-200c | GSE38817 | M | MSMC/M537 | GPL10558 |
| miR-200c | GSE38817 | M | LSMC/L537 | GPL10558 |
| miR-374a | GSE39356 | M | MCF7 | GPL6480 |
| miR-145 | GSE40387 | M | mesenchymal stem cells/Transfected using electroporation and synthetic negative control miR | GPL10558 |
| miR-145 | GSE40387 | M | mesenchymal stem cells/Transfected using lipofectamine and synthetic negative control miR | GPL10558 |
| let-7a | GSE6474 | M | A549 | GPL3050 |
| let-7c | GSE6838 | M | DLD1 | GPL3991 |
| let-7c | GSE6838 | M | HCT116 | GPL3991 |
| miR-17 | GSE6838 | M | DLD1 | GPL3991 |
| miR-17 | GSE6838 | M | HCT116 | GPL3991 |
| miR-20 | GSE6838 | M | DLD1 | GPL3991 |
| miR-20 | GSE6838 | M | HCT116 | GPL3991 |
| miR-200b | GSE6838 | M | HCT116diceR | GPL3991 |
| miR-34a | GSE7864 | M | A549 H1-teRm t | GPL4372 |
| miR-34a | GSE7864 | M | A549_p53 | GPL4372 |
| miR-34a | GSE7864 | M | DLD | GPL4372 |
| miR-34a | GSE7864 | M | HCT116 | GPL4372 |
| miR-34a | GSE7864 | M | HELA | GPL4372 |
| miR-34a | GSE7864 | M | TOV21G | GPL4372 |
| miR-34a | GSE7864 | M | TOV21G_p53 | GPL4372 |
| miR-34b | GSE7864 | M | A549 | GPL4372 |
| miR-34b | GSE7864 | M | A549_p53 | GPL4372 |
| miR-34b | GSE7864 | M | DLD | GPL4372 |
| miR-34b | GSE7864 | M | HCT116 | GPL4372 |
| miR-34b | GSE7864 | M | HELA | GPL4372 |
| miR-34b | GSE7864 | M | TOV21G | GPL4372 |
| miR-34b | GSE7864 | M | TOV21G_p53 | GPL4372 |
| miR-34c | GSE7864 | M | A549 | GPL4372 |
| miR-34c | GSE7864 | M | A549_p53 | GPL4372 |
| miR-34c | GSE7864 | M | DLD | GPL4372 |
| miR-34c | GSE7864 | M | HCT116 | GPL4372 |
| miR-34c | GSE7864 | M | HELA | GPL4372 |
| miR-34c | GSE7864 | M | TOV21G | GPL4372 |
| miR-34c | GSE7864 | M | TOV21G_p53 | GPL4372 |
| miR-155 | GSE9264 | M | kidney cells | GPL570 |
| miR-373 | GSE9742 | M | MCF7 | GPL6171 |
| miR-520c | GSE9742 | M | MCF7 | GPL6171 |
